# The mitigating effect of Spirulina (*Arthrospira platensis*) on the hemotoxicity of gibberellic acid on juvenile tilapia (*Oreochromis niloticus*)

**DOI:** 10.1007/s11356-022-23844-6

**Published:** 2022-11-08

**Authors:** Alaa El-Din H. Sayed, Mohamed Hamed, Abdelaziz A. A. El-Sayed, Bruno Nunes, Hamdy A. M. Soliman

**Affiliations:** 1 Zoology Department, Faculty of Science, Assiut University, Assiut, 71516 Egypt; 2 Zoology Department, Faculty of Science, Al-Azhar University, Assiut Branch, Assiut, 71524 Egypt; 3 Zoology Department, Faculty of Science, Zagazig University, Zagazig, 44519 Egypt; 4 Zoology Department, Faculty of Science, Islamic University of Madinah, Medina, 42238 Saudi Arabia; 5 Centro de Estudos Do Ambiente E Do Mar (CESAM), Universidade de Aveiro, Campus de Santiago, 3810-193 Aveiro, Portugal; 6 Departamento de Biologia, Universidade de Aveiro, Campus de Santiago, 3810-193 Aveiro, Portugal; 7 Zoology Department, Faculty of Science, Sohag University, Sohag, 8562 Egypt

**Keywords:** GA_3_, Comet assay, Poikilocytosis, Tilapia, Spirulina

## Abstract

The use of plant growth regulators has led to environmental contamination of water bodies that occur adjacent to agricultural areas. Some of these chemicals are bioactive, not only to plants, but also to non-target exposed biota, namely of the aquatic compartment. Previous work demonstrated the establishment of hepato- and nephrotoxic effects in juvenile tilapia (*Oreochromis niloticus*) exposed via aquatic media to gibberellic acid (GA_3_), which is among the most used plant growth regulators, in agricultural practices. Here, we investigated the effect of GA_3_ on hematological indices, poikilocytosis, nuclear abnormalities, and genotoxic indices measured in Nile tilapia (*Oreochromis niloticus*), as well as the putative protective effects of dietary supplementation of Spirulina (*Arthrospira platensis*). Fish were evenly assorted into 5 groups: group I served as a control, and groups II–V were fed diets supplemented with Spirulina at rates of 0 g/kg, 5 g/kg, 20 g/kg, and 100 g/kg, respectively, for 2 months before being exposed to 150 mg/L GA_3_. The results revealed that GA_3_ exposure decreased significantly all hematological indices (*P* < 0.05), except leucocytes and mean corpuscular hemoglobin concentration (MCHC), compared to the control group (*P* > 0.05). GA_3_ exposure increased significantly the percentage of nuclear abnormalities, altered erythrocytes and the percentages of tail DNA, compared to the control group (*P* < 0.05). Spirulina supplementation restored the hematological, poikilocytosis, nuclear abnormalities, and the percentages of tail DNA to near normal levels. The 100 g/kg SP treatment was the most effective in attaining such effect, showing concentration-dependency. The present study reinforces our findings of the toxicity of GA_3_ on *O. niloticus* and suggests that the addition of Spirulina to fish diet can mitigate the hemotoxic effects of GA_3_.

## Introduction

Gibberellic acid (GA_3_) is a widely used plant growth regulator (PGR) in agriculture, as it regulates fruit ripening and shoot growth (Taiz & Zeige 1991; Gianfagna 1995). However, agricultural companies often provide more GA_3_ than crops can uptake (Mickel 1978). When GA_3_ is applied by conventional air-assisted spraying, it can drift from the application location(Wei et al. 2016), thereby contaminating bodies of water via run-off or off-target spraying. Despite this possibility, and of its significant agricultural use, GA_3_ ecotoxicological profile has not been thoroughly studied so far, and a very limited amount of data on its final environmental fate, concentrations in the aquatic compartment, and specially, toxicological effects are still extremely scarce (EFSA 2012).

However, previous studies have determined that GA_3_ is not exempt of toxicity. The hematotoxicity of GA_3_ has been studied using animal models such as rats and mice (Tuluce and Çelik 2006; Muthu et al. 2011; Troudi et al. 2012a; Ali et al. 2021, 2022; Ahmed and Nofal 2021; Galal et al. 2021; Soliman et al. 2021a, b, 2022a, b; Bushra and Shenouda 2022), hens and quail hens (Elsebai et al. 2003b; Elkomy et al. 2008; Ismail 2009), and rabbits (El-Sebai 2004). Nevertheless, the impacts of GA_3_ on the hematological parameters and erythron profile of fish have not been investigated (Sayed et al. 2020). In fish, direct contact between highly vascularized structures, such as gills and polluted water represents one of the main exposure routes for contaminants to enter the bloodstream (Barboza et al. 2020). Accordingly, erythrocytes may provide a unique tool for cytotoxicity assessment in fish (Fazio 2019; Soliman & Sayed 2020). The hematological and erythron profiles (poikilocytosis, nuclear abnormalities) of fish erythrocytes are valuable biomarkers for assessing the toxicity of chemicals and pharmaceutical residues ( Sayed et al. 2013; Sayed et al. 2016). Comet assay or alkaline single-cell gel electrophoresis (SCGE) is a simple, fast, and sensitive technique for assessing genotoxicity by quantifying the amount of DNA damage in individual cells. Thus, SCGE is an important tool for environmental monitoring, and for assessing the health of aquatic species (Hamed et al. 2019a). Toxicological effects caused by exposure to GA_3_ may have a significant importance when considering fish species of high economic importance that are frequently cultured in the vicinity of agricultural areas. Due to this factor, some fish species (e.g., Nile tilapia, *Oreochromis niloticus*) are particularly prone to have their physiology challenged, following exposure to chemicals used in agricultural practices, such as PGRs.

The blue-green alga *Spirulina platensis* (SP) is a suitable and cost-effective natural antioxidant and immune-stimulant for humans and animals, with fewer side effects than synthetic products (Belay 2020). Spirulina contains vital compounds, including protein (50–70% on dry mass basis) with all essential amino acids (Farag et al. 2016), carotenoids, chlorophyll, pigments, essential fatty acids (alpha-linolenic, gamma-linolenic, and linoleic acid (Mendes et al. 2003, Peiretti and Giorgia 2011), photosynthetic pigments (Bermejo et al. 2008), vitamins (thiamine, nicotinamide, riboflavin, folic acid, pyridoxine, vitamins A, D, and E) (Hosseini et al. 2013) and minerals (such as Ca, K, Cr, Cu, Mn, Fe, P, Mg, Na, Zn, and Se) (Babadzhanov et al. 2004), making Spirulina an efficient feed supplement (Yousefi et al. 2019). Nevertheless, the potential remediation effects of Spirulina against toxicity of phytohormones such as GA_3_ have not been investigated, especially in fish of high economic importance (Sayed et al. 2020).

The Nile tilapia (*O. niloticus*) is one of the most economically important freshwater fishes of Egypt and has recently been used as a toxicological model in bioremediation studies (Sayed et al. 2015). Considering that the culture of Nile tilapia is often subjected to anthropogenic contamination, namely by agricultural xenobiotics, it is extremely important to understand the toxic effects deriving from such exposure and also how to prevent and revert the toxic effects caused by such chemicals. This is important, not only in purely economic terms (by preventing losses due to intoxication of cultured fish), but also ecologically. In this study, we investigated the protective effect of Spirulina supplementation on hematological and comet assay indices, as well as poikilocytosis, and nuclear abnormalities in late juvenile Nile tilapia exposed to gibberellic acid.

## Materials and methods

### Chemical and microalgae

Gibberellic acid (GA_3_) 10% was purchased from Jiangxi New Reyphon Biochemical Co., China. Spirulina tablets were bought from Japan Algae Co., Ltd. Spirulina tablets were dissolved in water, and their bioactive ingredients became available in the water column for fish to absorb via the gastrointestinal canal.

### Fish exposure

Early juveniles of tilapia (*O. niloticus*) (1 month old; weight 4.68 ± 0.1 g, length 3.45 ± 0.14 cm) were donated from an aquaculture unit (Al-Azhr University, Assiut) and transported to a laboratory in the Zoology Department of Assiut University, Egypt. The fish were kept in ≈200-L glass tanks (92 cm × 46 cm × 46 cm) containing 100 L dechlorinated tap water, with continuous aeration, under laboratory conditions for 4 weeks for acclimatization, and were fed 30% protein (Skerting Company®) diets.

The physicochemical properties of test water were recorded as follows: conductivity 269.5 μ M cm^−1^, pH 7.4, dissolved oxygen 6.85 mg L^−1^, temperature 20.9 °C, photoperiod 12 h:12 h light:dark. Five groups (30 fish/group) were assigned in three replicates for each treatment group (10 fish/glass aquarium) during the experimental period; group I served as a control, and groups II–V were fed 30% protein (Skerting Company®) diets supplemented with SP at rates of 0 g/kg, 5 g/kg, 20 g/kg, and 100 g/kg, respectively, for 2 months. These levels of supplementation were based on previously obtained data (Sayed & Fawzy 2014). Fish were subjected to these conditions before being exposed to 150 mg/L GA_3_ (Boeri 1991). Animals were physically separated from each other, in independent aquaria.

After 15 days, 6 fish from each group were randomly selected and anesthetized using ice to reduce the stress during processing. This procedure has been proved to be rapid, effective, and less stressful for animals, not causing significant changes in the structures of sampled tissues (Wilson et al. 2009). Blood was collected from the caudal vein in heparinized tube for hematological, comet assay, poikilocytosis, and nuclear abnormality analyses.

### Hematological parameters

The hematological parameters, red blood cells (RBCs), white blood cells (WBCs), platelets, hematocrit (Ht), hemoglobin (Hb), mean corpuscular volume (MCV), mean corpuscular hemoglobin (MCH), and MCH concentration (MCHC), were assessed according to Fijan (2002).

### Erythron profile (poikilocytosis and nuclear abnormalities of RBCs)

Blood smears were prepared, stained with hematoxylin-eosin, selected, coded, randomized, and blindly scored for erythrocyte alterations and nuclear abnormalities, according to the criteria of al-Sabti and Metcalfe (1995) and Schmid (1975).

### Comet assay

At the end of the experiment, blood (50 μL) was collected from the caudal veins, according to Sayed et al. (2017), then immediately placed on ice to prevent endogenous DNA damage and repair in the unfixed cells. Cell viability was assessed using a hemocytometer. A neutral comet assay was conducted using a previously described protocol (Hidaka et al. 2010) with minor modifications (Sayed et al. 2017). Cells were observed under a Zeiss Axioplan 2 fluorescence microscope (200×) with a digital 3 CCD color video camera (Sony, AVT-Horn). CASP software was used to analyze the comet image and calculate the tail moment score for each cell (Końca et al. 2003).

### Statistical analyses

Data were analyzed using SPSS (version 25) at a significance level of 0.05. Briefly, data were

tested for normality using the Shapiro–Wilk test. Then, data were tested for homogeneity of variances (Levene’s test) using one-way analysis of variance. In the case of variance equality, Fisher’s LSD post hoc test was used to compare treatment groups against the control; in cases of variance inequality, Dunnett’s post hoc test was used.

### Ethical statement

Experimental design and fish treatment were approved by the Committee of the Faculty of Science, Assuit University.

## Results

### Hematological parameters

The results of the current work revealed that the hematological indices, erythrocyte count (RBCs), Hb, Ht, MCH, MCV, and platelet levels, were significantly decreased in group II (exposed to GA_3_), compared to the control group. However, these levels were significantly increased in groups treated with SP (group III, IV, and V), compared to group II (Table 1). Conversely, there was an increase in MCHC and WBC levels in group II (exposed to GA_3_), compared with the control group; these parameters significantly decreased in groups treated with SP, compared with group II, reaching normal levels.

**Table 1:** Hematological indices of juvenile Nile tilapia (*Oreochromis niloticus*) exposed to 150 mg/L gibberellic acid and previously fed diets supplemented with *Spirulina platensis*

	1^st^ group	2^nd^ group	3^rd^ group	4^th^ group	5^th^ group
(RBC) (million/mm^3^)	1.9 ± 0.0^a^	1.7 ± 0.0^b^	1.8 ± 0.0^c^	1.9 ± 0.0^d^	1.9 ± 0.0^a^
Hemoglobin (Hb) (g/dL)	8.7 ± 0.1^a^	8.1 ± 0.0^b^	8.1 ± 0.0^b^	8.4 ± 0.0^c^	8.5 ± 0.1^c^
Ht (PCV) (%)	26.3 ± 0.3^a^	23.3 ± 0.2^b^	24.7 ± 0.2^c^	24.7 ± 0.2^c^	26.5 ± 0.1^a^
MCV (µm^3^)	135.1 ± 1.2^ab^	134.2 ± 2.5^ab^	136.03 ± 1.2^ab^	133.5 ± 1.2^a^	138.7 ± 1.4^b^
MCH (Pg)	44.5 ± 0.5^a^	46.3 ± 0.5^b^	45 ± 0.1^a^	45.03 ± 0.1^a^	44.4 ± 0.6^a^
MCHC (%)	33 ± 0.4^ac^	34.5 ± 0.3^b^	33 ± 0.3^a^	34 ± 0.3^b^	32.01 ± 0.3^c^
Platelets (Thousands/mm^3^)	317.7 ± 0.5^a^	311.5 ± 0.2^b^	313 ± 0.4^c^	314 ± 0.4^c^	315.7 ± 0.4^d^
(WBC) (thousands/mm^3^)	856 ± 1.9^a^	861.5 ± 2.1^b^	850.2 ± 1.4^ cd^	853.5 ± 0.9^ac^	848 ± 1.8^d^

Data are represented as mean ± SE. Values with different superscript letters in the same row are significantly different (*P* < 0.05). Treatments consisted of exposure to 150 mg/L gibberellic acid (GA_3_) and/or diet supplementation with *Spirulina platensis* (SP) for 2 months prior. Group I, control group; group II, 150 mg/L GA_3_; group III, 150 mg/L GA_3_ and 5 g/kg SP diet; group IV, 150 mg/L GA_3_ and 20 g/kg SP diet; group V, 150 mg/L GA_3_ and 100 g/kg SP diet. *RBC* red blood cells; *H*t hematocrit; *PCV* packed-cell volume; *Hb* hemoglobin; *MCV* mean corpuscularvolume; *MCH* mean corpuscular hemoglobin; *MCHC* mean corpuscular hemoglobin concentration; *WBC* white blood cells

### Poikilocytosis (morphological changes of erythrocytes)

**Fig. 1:**
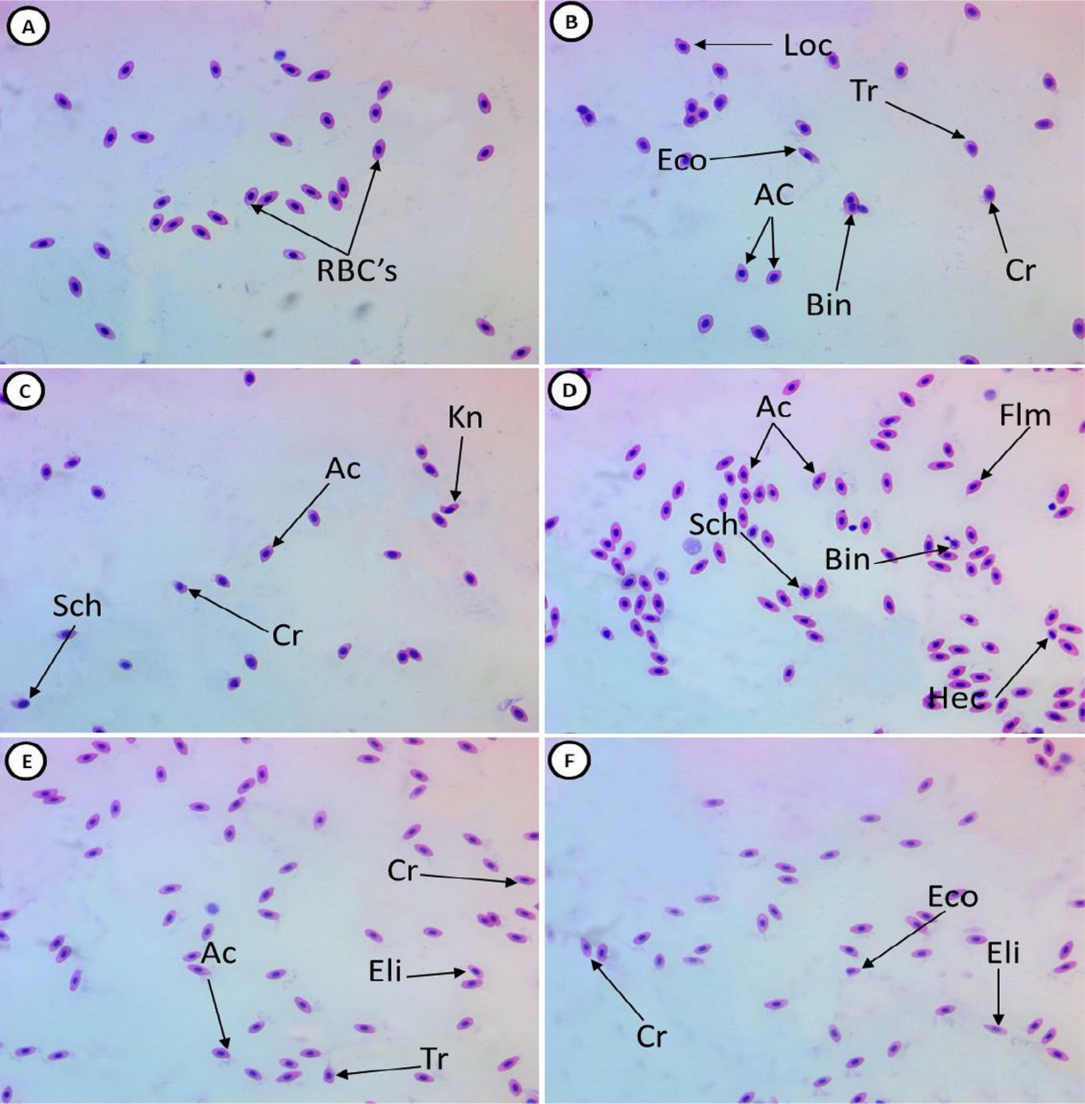
Representative hematoxylin–eosin-stained blood smears collected from juvenile Nile tilapia (*Oreochromis niloticus*) showing normal erythrocytes (**A**), deformed erythrocytes after fish exposure to 150 mg/L gibberellic acid (GA_3_) for 15 days (**B**, **C**), deformed erythrocytes after fish exposure to 150 mg/L GA_3_ for 15 days and feed supplemented with 5 g/kg *Spirulina platensis* (SP) for 2 months (**D**), deformed erythrocytes after fish exposure to 150 mg/L GA_3_, and 20 g/kg SP diet (**E**), and deformed erythrocytes after exposure to 150 mg/L GA_3_, and 100 g/kg SP diet (**F**). Tr, tear-drop cell; Cr, crenated cell; Ac, acanthocyte; Eco, eccentric nucleus; Hec, hemolyzed cells; Bin, bionuclei; Loc, loped cell; Eli, elliptocyte; Shc, schistocyte; Kn, kidney cell (1000 × magnification)

Our results showed that, compared to the control, exposure to GA_3_ resulted in a significant increase in the percentage of RBC morphological changes, as well as the appearance of some pathologic cells (Table 2; Fig. 1b and c), including tear-drop cells, crenated cells, acanthocytes, eccentric nuclei, hemolyzed cells, bionuclei, loped cells, elliptocytes, schistocytes, and kidney cells. The prevalence of these changes was significantly reduced in groups pre-fed SP, compared to group II (Table 2; Fig. 1d, e, and f).

**Table 2:** Percentage of altered erythrocytes of juvenile Nile tilapia (*Oreochromis niloticus*) exposed to 150 mg/L gibberellic acid and previously fed diets supplemented with *Spirulina platensis*

	1^st^ group	2^nd^ group	3^rd^ group	4^th^ group	5^th^ group
Hemolyzed cell	2.33 ± 0.33^a^	12.33 ± 0.67^b^	8.33 ± 0.33^c^	7.67 ± 0.88^c^	4.67 ± 0.33^d^
Sickle cell	0.67 ± 0.33^a^	5.66 ± 0.33^b^	4.33 ± 0.33^b^	2.66 ± 0.33^c^	1.66 ± 0.33^ac^
Irregular cell	0 ± 0.00^a^	16.33 ± 1.20^b^	12.33 ± 0.33^c^	7.33 ± 0.33^d^	3.33 ± 0.88^e^
Schistocyte	0.33 ± 0.33^a^	13.66 ± 0.88^b^	11.33 ± 0.88^c^	7.33 ± 0.67^d^	5.33 ± 0.33^d^
Acanthocyte	1.33 ± 0.33^a^	13 ± 1.15^b^	10.33 ± 0.66^b^	8 ± 0.57^b^	3.66 ± 0.33^c^
Tear drop	0.33 ± 0.33^a^	12.33 ± 0.67^b^	8.33 ± 0.67^bc^	5 ± 0.58^ cd^	3.33 ± 0.33^d^
Heinz bodies	0.67 ± 0.67^a^	5.67 ± 1.2^bc^	3.67 ± 0.33^c^	2.67 ± 0.67^ac^	1.33 ± 0.33^a^
Elliptocyte	1.33 ± 0.33^a^	12.33 ± 0.88^b^	10.33 ± 0.67^c^	6.67 ± 0.33^d^	3.33 ± 0.67^e^
Heart shape	0 ± 00^a^	4.67 ± 0.88^b^	2.33 ± 0.33^c^	0.67 ± 0.33^a^	0.33 ± 0.33^a^
Eccentric nucleus	2 ± 0.57^a^	14 ± 0.57^b^	12 ± 0.57^c^	8.67 ± 0.33^d^	3.67 ± 0.33^e^
Crenated cell	2.33 ± 0.33^a^	23.33 ± 0.88^b^	17 ± 0.57^c^	12.33 ± 0.33^d^	5.67 ± 0.33^e^
Kidney-shape	0 ± 0.00^a^	4 ± 0.57^b^	1.67 ± 0.33^c^	1.33 ± 0.33^c^	0.67 ± 0.33^ac^
Total malformed	11.33 ± 3.30^a^	137 ± 1.50^b^	102 ± 1.00^c^	70.33 ± 2.90^d^	37 ± 2.60^e^

Data are represented as mean ± SE. Values with different superscript letters in the same row are significantly different (*P* < 0.05). Treatments consisted of exposure to 150 mg/L gibberellic acid (GA_3_) and/or diet supplementation with *Spirulina platensis* (SP) for 2 months prior. Group I, control group; group II, 150 mg/L GA_3_; group III, 150 mg/L GA_3_ and 5 g/kg SP diet; group IV, 150 mg/L GA_3_ and 20 g/kg SP diet; group V, 150 mg/L GA_3_ and 100 g/kg SP diet

### Erythrocyte nuclei alterations

Compared to the control group, fish exposed to GA_3_ exhibited a significant increase in the percentage of erythrocyte nuclei alterations (Table 3; Fig. 1b and c), including micronuclei, binucleated, blebbed nuclei, notched nuclei, lobed nuclei, and hemolyzed nuclei. These alterations were significantly reduced in groups pre-fed SP, compared to group II (Table 3; Fig. 1d, e, and f).

**Table 3:** Percentage of altered erythrocyte nuclei of juvenile Nile tilapia (*Oreochromis niloticus)* exposed to 150 mg/L gibberellic acid and previously fed diets supplemented with *Spirulina platensis*

	1^st^ group	2^nd^ group	3^rd^ group	4^th^ group	5^th^ group
Micronuclei	0.67 ± 0.33^a^	11.33 ± 0.33^b^	8.66 ± 0.33^c^	5.66 ± 0.88^d^	3.33 ± 0.33^e^
Binucleated	0.67 ± 0.33^a^	10 ± 0.58^b^	7.33 ± 0.33^c^	4.33 ± 0.33^d^	3.33 ± 0.33^d^
Blebbed nuclei	0 ± 0.00^a^	2.33 ± 0.33^b^	1.67 ± 0.33^bc^	1.33 ± 0.33^c^	0.67 ± 0.33^ac^
Notched nuclei	0.33 ± 0.33^a^	4.66 ± 0.67^b^	2.33 ± 0.33^c^	1.67 ± 0.33^c^	1.33 ± 0.33^ac^
Lobed nuclei	0 ± 0.00^a^	2.33 ± 0.67^b^	1.33 ± 0.33^bc^	1 ± 0.00^c^	0.33 ± 0.33^ac^
Hemolyzed nuclei	0.67 ± 0.33^a^	6.67 ± 0.33^b^	5 ± 0.58^c^	3.67 ± 0.33^d^	2.33 ± 0.33^e^
Total malformed	2.33 ± 0.33^a^	37.33 ± 0.33^b^	26.33 ± 0.88^c^	17.66 ± 1.45^d^	11.33 ± 0.33^e^

Data are represented as mean ± SE. Values with different superscript letters in the same row are significantly different (*P* < 0.05). Treatments consisted of exposure to 150 mg/L gibberellic acid (GA_3_) and/or diet supplementation with *Spirulina platensis* (SP) for 2 months prior. Group I, control group; group II, 150 mg/L GA_3_; group III, 150 mg/L GA_3_ and 5 g/kg SP diet; group IV, 150 mg/L GA_3_ and 20 g/kg SP diet; group V, 150 mg/L GA_3_ and 100 g/kg SP diet

### Comet assay

**Fig. 2:**
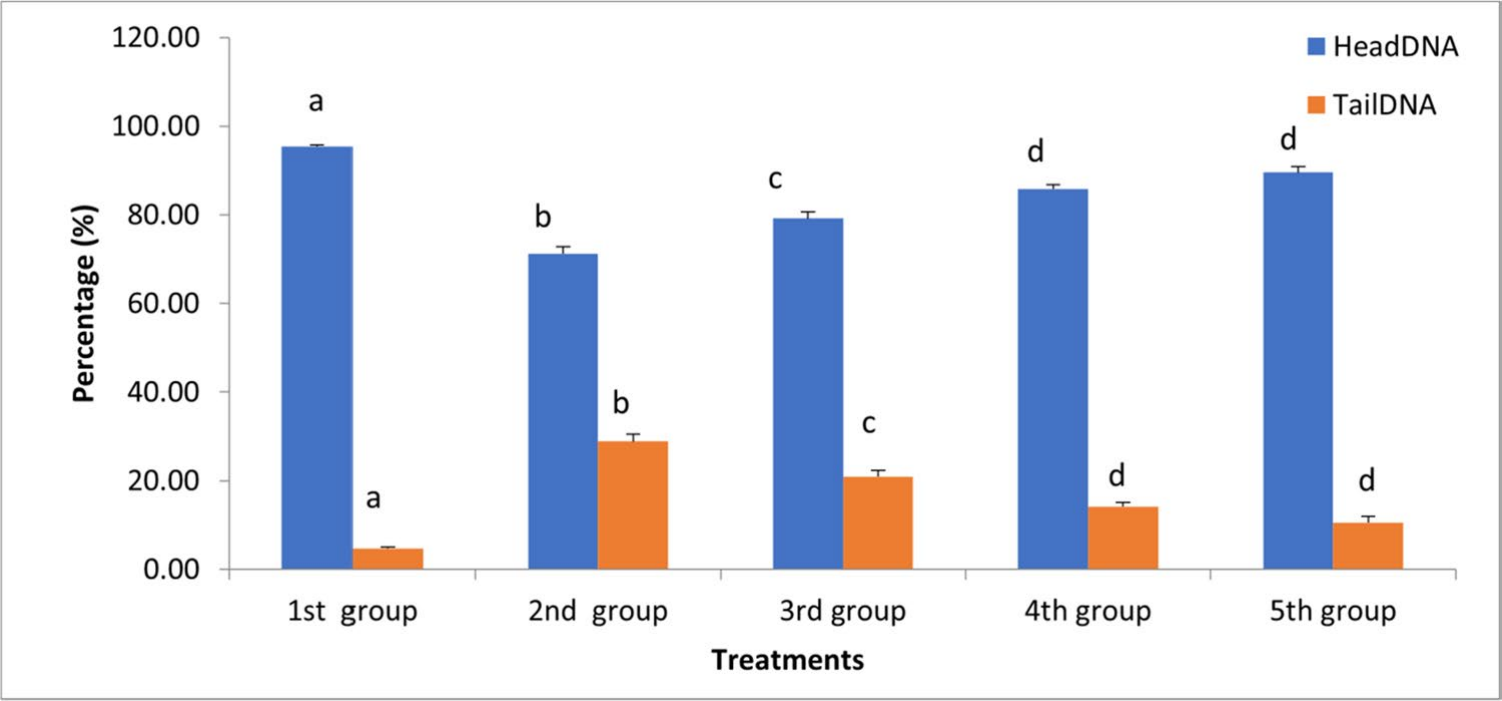
Percentages of head DNA and tail DNA in blood cell comet assays of juvenile Nile tilapia (*Oreochromis niloticus*). Treatments consisted of exposure to 150 mg/L gibberellic acid (GA_3_) and/or diet supplementation with *Spirulina platensis* (SP) for 2 months prior. Group I, control group; group II, 150 mg/L GA_3_; group III, 150 mg/L GA_3_ and 5 g/kg SP diet; group IV, 150 mg/L GA_3_ and 20 g/kg SP diet; group V, 150 mg/L GA_3_ and 100 g/kg SP diet.

**Fig. 3:**
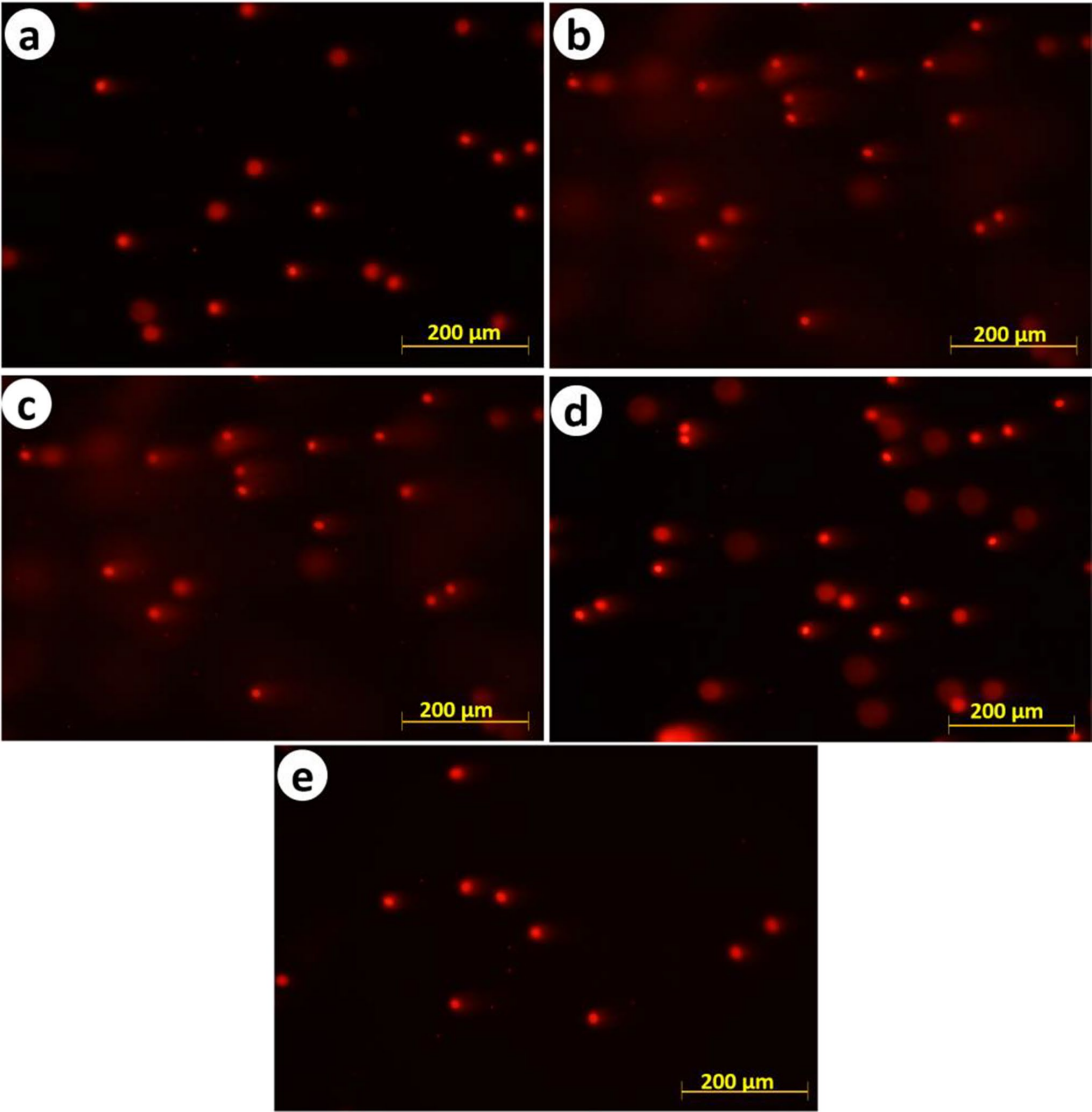
Blood cells stained with ethidium bromide after comet assay from control and treated groups of juvenile Nile tilapia (*Oreochromis niloticus*). Treatments consisted of exposure to 150 mg/L gibberellic acid (GA_3_) and/or diet supplementation with *Spirulina platensis* (SP) for 2 months prior. **A** Group I, control group; **B** group II, 150 mg/L GA_3_; **C** group III, 150 mg/L GA_3_ and 5 g/kg SP diet; **D** group IV, 150 mg/L GA_3_ and 20 g/kg SP diet; **E** group V, 150 mg/L GA_3_ and 100 g/kg SP diet. Scale bar 25 μm

In comparison with the control group, a clear and statistically significant increase in the percentages of tail DNA and decrease in head DNA was found in group II, treated with GA_3_ (Fig. 2). Undamaged control erythrocytes showed comets with an intact head (Fig. 3a), while the results of the comet assay of fish from group II showed an increase in tail length, compared to the control group (Fig. 3b). Pre-feeding with SP attenuated the changes observed in group II, but the number of alterations in groups III–V was still higher than those observed for the control group (Figs. 2; 3c, d, and e).

## Discussion

Although gibberellic acid is extensively used as a plant growth regulator in Egypt and other countries, little is known about the toxic effects of GA_3_ on fish (Sayed et al. 2020). Hematological parameters are important measures that reflect fish health status (Thummabancha et al. 2016). The results of the current work revealed that erythrocyte count (RBCs), Hb, Ht, MCH, MCV, and platelet levels, were significantly decreased in GA_3_-exposed fish group. Additionally, El-Okazy (2008) observed significant dose-dependent decreases in total erythrocyte counts and Hb levels in mice treated with gibberellic acid; total leukocyte counts showed a highly significant dose-dependent increase. Elkomy et al. (2008) reported that GA_3_ doses caused a significant increase in Hb concentration and packed-cell volume (PCV), but RBC count was not significantly increased in aged female fowl. Furthermore, Troudi et al. (2012a) stated that GA_3_ reduced the RBC count, Hb concentration, and Ht in suckling rats, while these parameters remained unchanged in their mothers. However, an increase in WBC count was observed in mothers but not their pups. In addition, Abdou et al. (2017) observed that direct exposure to GA_3_ for 1 month in rams induced a significant decrease in blood picture.

In contrast, Zilva and Pannall (1983) noted that GA_3_ affected PCV in birds by increasing hematopoiesis and subsequently increasing erythrocyte counts. Similarly, Abdelhamid et al. (1994) reported that GA_3_ treatment increased the plasma iron concentration in chicks; the improved Hb concentration in chicks seems to be due to increased iron absorption rates from the diet. In addition, Ozmen et al. (1995) demonstrated that GA_3_ treatment significantly increased erythrocyte counts in laboratory mice. Elsebai et al. (2003a) reported significant dose-dependent increases in quail hen RBC, Hb, and PCV% after GA_3_ exposure. Elsebai (2004) revealed that GA_3_ significantly increased RBC count, WBC count, PCV, and Hb in rabbits in a dose-dependent manner. Srikumar et al. (2009) reported that GA_3_ influenced changes in RBC and WBC content and increased Hb in rats. Ismail (2009) found that RBCs and Hb were significantly increased in laying hens injected with GA_3_, compared with the control group. In male albino rats, RBC, WBC, and neutrophil counts significantly increased at all doses of GA_3_ treatment, possibly due to the effect of GA_3_ on hematopoiesis, whereas lymphocyte counts decreased (Muthu et al. 2011). Askar and IsmaeI (2012) found that WBCs, RBCs, PCV, and lymphocytes improved significantly in hens injected with GA_3_.

Despite the lack of knowledge concerning the mechanisms by which GA_3_, and PGRs in general, exert their adverse effects, it may be suggested that oxidative stress may be a decisive event. PGRs may induce oxidative stress, leading to generation of free radicals and cause lipid peroxidation, which causes changes in fluidity and an increase in the permeability to different ions leading to hemolysis of RBCs (Tuluce & Çelik 2006). The data obtained by Tuluce and Celike (2006) showed that GA_3_ induced alterations in antioxidant defense systems and lipid peroxidation level in erythrocytes in rats exposed to 75 ppm of GA_3_ in drinking water for 50 days. In addition, Troudi et al. (2011a,b; 2012b) showed that GA_3_ was able to induce significant oxidative changes in rodents, with the activation of antioxidant defensive mechanisms, followed also by an increase in lipid peroxidation. Oxidative stress was again the main outcome resulting from GA_3_ exposure, in the insect species *Galleria mellonella* L., with the activation of a series of antioxidant mechanisms, such as superoxide dismutase, glutathione S-transferase, and catalase, as demonstrated by Altuntas (2015). This effect of favoring oxidative stress seems not be limited to animals, since sorghum (*Sorghum bicolor*), after being exposed to GA_3_, also had changes in the levels of antioxidant enzymes, as described by Forghani et al. (2020). However, the role of GA_3_ in the modulation of antixodiant responses in plants is not absolutely straightforward and will depend on the existence of other sources of physiological stress. The administration of GA_3_ to plants (maize, *Zea mays*) subjected to salinity stress resulted in the increase of the efficacy of antioxidant defense, namely by augmenting the activity of antioxidant enzymes (Shahzad et al. 2021). In addition, antioxidant enzymes (SOD, and CAT) were significantly elevated in GA_3_-treated tilapia (Sayed et al. 2020).

The results of the present study demonstrated that fish exposure to GA_3_ resulted in significant increases in poikilocytosis (RBC morphological changes) and the appearance of some pathologic cells and nuclei. Similarly, Troudi et al. (2012a) used blood smear analyses to assess GA_3_-treated rat pups and observed empty red cells, which is a sign of anemia; the mothers’ blood smears showed polynuclear blood cell infiltration. Also, Ayoub and El Aalem (2016) observed that GA_3_-exposed groups evoked a significant increase in the total aberrated cells and total chromosomal aberrations of bone marrow cells in rabbit.

To the best of our knowledge, this is the first study to determine the effect of GA_3_ on RBC poikilocytosis and nuclear abnormalities. A growing amount of evidence indicates that GA_3_ alters the antioxidative systems as it induces oxidative stress, leading to generation of free radicals and causes lipid peroxidation which may trigger RBC membrane damage, such as a decreased membrane transporter activity and alterations in membrane permeability (Troudi et al. 2012a). This effect may be the source of cellular damage that ultimately results in morphological changes on blood cells. In fact, previously published data have established the linkage between the occurrence of oxidative stress (and of its concomitant adverse changes, including oxidation of membrane lipids and proteins) and morphological changes in erythrocytes (Gyawali et al. 2015a), which in turn, alters blood viscosity parameters (Gyawali and Richards 2015b). In fact, oxidative stress seems to be a major force in deleterious changes to the morphology of erythrocytes, as evidenced by the review by Bissinger et al. (2018).

Comet assays are used to detect genetic damage in the form of DNA strand breaks, providing a sensitive indicator of changes in the overall health of an organism. Comet assays have been applied to assess and monitor the health and genetic condition of both vertebrate and invertebrate organisms (Hamed et al. 2019b). Blood is readily available and easy to collect from fish; RBCs comprise 97% of fish blood. Hence, fish RBCs are frequently used to evaluate DNA damage using comet assay (Tasneem &Yasmeen 2018). The DNA damage observed in the blood cells in the present study was similar to that of Hosseinchi et al. (2013) who stated that GA_3_ increased the immature sperms and sperms with damaged chromatin. Also, DNA isolated from control and tadpoles treated with gibberellin-A_3_ showed degradation into oligonucleotide fragments forming a clear laddering pattern of apoptosis (Sakr and Shalaby 2012). Substances that induce genotoxicity have been shown also to produce reactive oxygen species, as well as electrophilic free-radical metabolites that interact with DNA and lead to DNA damage (Sayed et al. 2020). It is likely that GA_3_ metabolism may result in the overproduction of ROS. This outcome has been already reported, and for a considerable number of animal models, namely rodents (Troudi et al. 2012a; Tuluce and Çelik 2006), as already described. Sakr et al. (2009) stated that by increasing the concentration of gibberellin A_3_, the number of damage cells and the damage DNA spots increase. Also, DNA fragmentation was markedly increased in the retina of treated mother rats and their offspring’s after intra-gastric administration of gibberellic and indole acetic acids (El-Sayyad et al. 2015). Consequently, it is possible to suggest that the here-observed DNA damage may be associated to the occurrence of oxidative stress, which is a common outcome of GA_3_ exposure in vertebrates.

The addition of *S. platensis* to the fish diets significantly restored hematological parameters and erythron profiles to their normal levels and shapes. These results are attributed to the bioactive components of *S. platensis*. Among these, it is possible to suggest that the combination of iron and vitamins, is particularly effective in preventing the hematological adverse effects caused by GA_3_. On one hand, iron is detrimental for the control of the production of red blood cells and decrease the RBC destruction that results from *O. niloticus* exposure to GA_3_ (Hemalatha et al. 2012). *S. platensis* also contains polysaccharides, which induce RBC regeneration (Mohamed et al. 2014). Furthermore, *S. platensis* contains phycocyanin pigment, which induces erythropoietin (EPO) hormone and contributes to erythropoiesis (Zhang 1994), and beta-carotene, which enhances RBC recovery to reduce cell lysis (Stivala et al. 1996). Also, the addition of SP to fish diet decreased the level of DNA damage induced by GA_3_ exposure. This could be ascribed to the antioxidant components of Spirulina, which scavenge free radicals to reduce oxidative DNA damage (Chu et al. 2010). Spirulina has been long known for its antioxidant activity (Piñero Estrada et al. 2001; Dartsch 2008) and has been shown to exert antioxidant activity on several animals model exposed to known oxidant chemicals, such as cadmium (Amin et al. 2006), sodium arsenite (Bashandy et al. 2016), deltamethrim (Abdel-Daim et al. 2013), glyphosate (Wided et al. 2021), and γ-irradiation and thioacetamide (Salem and Ismail 2021). Spirulina has also been implicated in the prevention of oxidative damage and inflammation resulting from excessive physical activity (Brito et al. 2020). In addition, *S. platensis* contains polysaccharides, which improve both the repair activity of damaged DNA excision and unscheduled DNA synthesis (Bhat and Madyastha 2001).

In conclusion, supplementing fish diets with SP can mitigate the adverse effects of gibberellic acid.

## Data Availability

All the data generated or analyzed during this study are included in the research article.
